# Supplementary material for: Variable Dimensionality of Europium(III) and Terbium(III) Coordination Compounds with a Flexible Hexacarboxylate Ligand
Source: Molecules. 2022 Nov 14;27(22):7849. doi: 10.3390/molecules27227849 (PMC9696389; doi:10.3390/molecules27227849)

checkCIF/PLATON report

Structure factors have been supplied for datablock(s) tbl3_sq

THIS REPORT IS FOR GUIDANCE ONLY. IF USED AS PART OF A REVIEW PROCEDURE FOR PUBLICATION, IT SHOULD NOT REPLACE THE EXPERTISE OF AN EXPERIENCED CRYSTALLOGRAPHIC REFEREE.

No syntax errors found. CIF dictionary Interpreting this report

Datablock: tbl3_sq

Bond precision:	C-C = 0.0030 A		Wavelength=0.71073
Cell:	a=38.585(2)	b=13.1992(7)	c=11.9356(7)
	alpha=90	beta=106.289(2)	gamma=90
Temperature:	150 K		

	Calculated	Reported
Volume	5834.7(6)	5834.6(6)
Space group	C 2/c	C 2/c
Hall group	-C 2yc	-C 2yc
Moiety formula	C60 H42 O35 Tb [+ solvent]	?
Sum formula	C60 H42 O35 Tb [+ solvent]	C60 H42 O35 Tb
Mr	1481.87	1481.85
Dx,g cm-3	1.687	1.687
Z	4	4
Mu (mm-1)	1.320	1.320
F000	2988.0	2988.0
F000'	2989.23	
h,k,lmax	56,19,17	56,19,17
Nref	9729	9710
Tmin,Tmax	0.906,0.962	0.668,0.746
Tmin'	0.818	

Correction method= # Reported T Limits: Tmin=0.668 Tmax=0.746
AbsCorr = MULTI-SCAN

Data completeness= 0.998 Theta(max)= 31.511

R(reflections)= 0.0353(8319) wR2(reflections)= 0.0786(9710)

S = 1.061 Npar= 463

The following ALERTS were generated. Each ALERT has the format
test-name_ALERT_alert-type_alert-level.
Click on the hyperlinks for more details of the test.

Alert level C

```
CRYSC01_ALERT_1_C The word below has not been recognised as a standard
            identifier.
            yellowish
CRYSC01_ALERT_1_C No recognised colour has been given for crystal colour.
PLAT213_ALERT_2_C Atom O13             has ADP max/min Ratio .....        3.8 prolat
PLAT220_ALERT_2_C NonSolvent   Resd 1  O   Ueq(max)/Ueq(min) Range        6.0 Ratio
PLAT222_ALERT_3_C NonSolvent Resd 1  H   Uiso(max)/Uiso(min) Range        9.4 Ratio
PLAT242_ALERT_2_C Low    'MainMol' Ueq as Compared to Neighbors of       Tb01 Check
PLAT245_ALERT_2_C U(iso) H1A       Smaller than U(eq) O20       by      0.018 Ang**2
PLAT245_ALERT_2_C U(iso) H1B       Smaller than U(eq) O20       by      0.018 Ang**2
PLAT975_ALERT_2_C Check Calcd Resid. Dens.  0.84Ang From O20     .       1.18 eA-3
PLAT975_ALERT_2_C Check Calcd Resid. Dens.  0.97Ang From O20     .       0.64 eA-3
PLAT975_ALERT_2_C Check Calcd Resid. Dens.  1.07Ang From O13     .       0.42 eA-3
PLAT976_ALERT_2_C Check Calcd Resid. Dens.  0.44Ang From O13     .      -0.53 eA-3
PLAT976_ALERT_2_C Check Calcd Resid. Dens.  0.67Ang From O13     .      -0.53 eA-3
PLAT977_ALERT_2_C Check Negative Difference Density on H1B       .      -0.33 eA-3
```

Alert level G

```
PLAT007_ALERT_5_G Number of Unrefined Donor-H Atoms .............          5 Report
PLAT128_ALERT_4_G Alternate Setting for Input Space Group     C2/c       I2/a Note
PLAT232_ALERT_2_G Hirshfeld Test Diff (M-X)  Tb01     --O2       .       13.4 s.u.
PLAT300_ALERT_4_G Atom Site Occupancy of O19       Constrained at         0.7 Check
PLAT300_ALERT_4_G Atom Site Occupancy of O18       Constrained at         0.3 Check
PLAT300_ALERT_4_G Atom Site Occupancy of H1C       Constrained at         0.5 Check
PLAT300_ALERT_4_G Atom Site Occupancy of H13C      Constrained at         0.5 Check
PLAT300_ALERT_4_G Atom Site Occupancy of H19A      Constrained at         0.7 Check
PLAT300_ALERT_4_G Atom Site Occupancy of H18A      Constrained at         0.3 Check
PLAT301_ALERT_3_G Main Residue  Disorder .............(Resd  1  )         2% Note
PLAT303_ALERT_2_G Full Occupancy Atom H100      with # Connections       2.00 Check
PLAT416_ALERT_2_G Short Intra D-H..H-D       H1       ..H1C      .       0.98 Ang.
                                                      x,y,z  =      1_555 Check
PLAT432_ALERT_2_G Short Inter X...Y Contact  O5       ..C2       .       2.96 Ang.
                                            3/2-x,1/2-y,1-z  =      7_656 Check
PLAT432_ALERT_2_G Short Inter X...Y Contact  O9       ..C29      .       2.96 Ang.
                                         3/2-x,-1/2+y,3/2-z  =      4_646 Check
PLAT605_ALERT_4_G Largest Solvent Accessible VOID in the Structure        179 A**3
PLAT720_ALERT_4_G Number of Unusual/Non-Standard Labels ..........          1 Note
PLAT767_ALERT_4_G INS Embedded LIST 6 Instruction Should be LIST 4     Please Check
PLAT869_ALERT_4_G ALERTS Related to the Use of SQUEEZE  Suppressed          ! Info
PLAT883_ALERT_1_G No Info/Value for _atom_sites_solution_primary .     Please Do !
PLAT910_ALERT_3_G Missing # of FCF Reflection(s) Below Theta(Min).          2 Note
PLAT912_ALERT_4_G Missing # of FCF Reflections Above STh/L=  0.600         18 Note
PLAT941_ALERT_3_G Average HKL Measurement Multiplicity ...........        4.8 Low
PLAT961_ALERT_5_G Dataset Contains no Negative Intensities .......     Please Check
PLAT978_ALERT_2_G Number C-C Bonds with Positive Residual Density.          8 Info
```

0 **ALERT level A** = Most likely a serious problem - resolve or explain
0 **ALERT level B** = A potentially serious problem, consider carefully

```
14 ALERT level C = Check. Ensure it is not caused by an omission or oversight
24 ALERT level G = General information/check it is not something unexpected

 3 ALERT type 1 CIF construction/syntax error, inconsistent or missing data
17 ALERT type 2 Indicator that the structure model may be wrong or deficient
 4 ALERT type 3 Indicator that the structure quality may be low
12 ALERT type 4 Improvement, methodology, query or suggestion
 2 ALERT type 5 Informative message, check
```

It is advisable to attempt to resolve as many as possible of the alerts in all categories. Often the minor alerts point to easily fixed oversights, errors and omissions in your CIF or refinement strategy, so attention to these fine details can be worthwhile. In order to resolve some of the more serious problems it may be necessary to carry out additional measurements or structure refinements. However, the purpose of your study may justify the reported deviations and the more serious of these should normally be commented upon in the discussion or experimental section of a paper or in the "special_details" fields of the CIF. checkCIF was carefully designed to identify outliers and unusual parameters, but every test has its limitations and alerts that are not important in a particular case may appear. Conversely, the absence of alerts does not guarantee there are no aspects of the results needing attention. It is up to the individual to critically assess their own results and, if necessary, seek expert advice.

Publication of your CIF in IUCr journals

A basic structural check has been run on your CIF. These basic checks will be run on all CIFs submitted for publication in IUCr journals (*Acta Crystallographica*, *Journal of Applied Crystallography*, *Journal of Synchrotron Radiation*); however, if you intend to submit to *Acta Crystallographica Section C* or *E* or *IUCrData*, you should make sure that full publication checks are run on the final version of your CIF prior to submission.

Publication of your CIF in other journals

Please refer to the *Notes for Authors* of the relevant journal for any special instructions relating to CIF submission.

PLATON version of 12/09/2022; check.def file version of 09/08/2022

Datablock tbl3_sq - ellipsoid plot

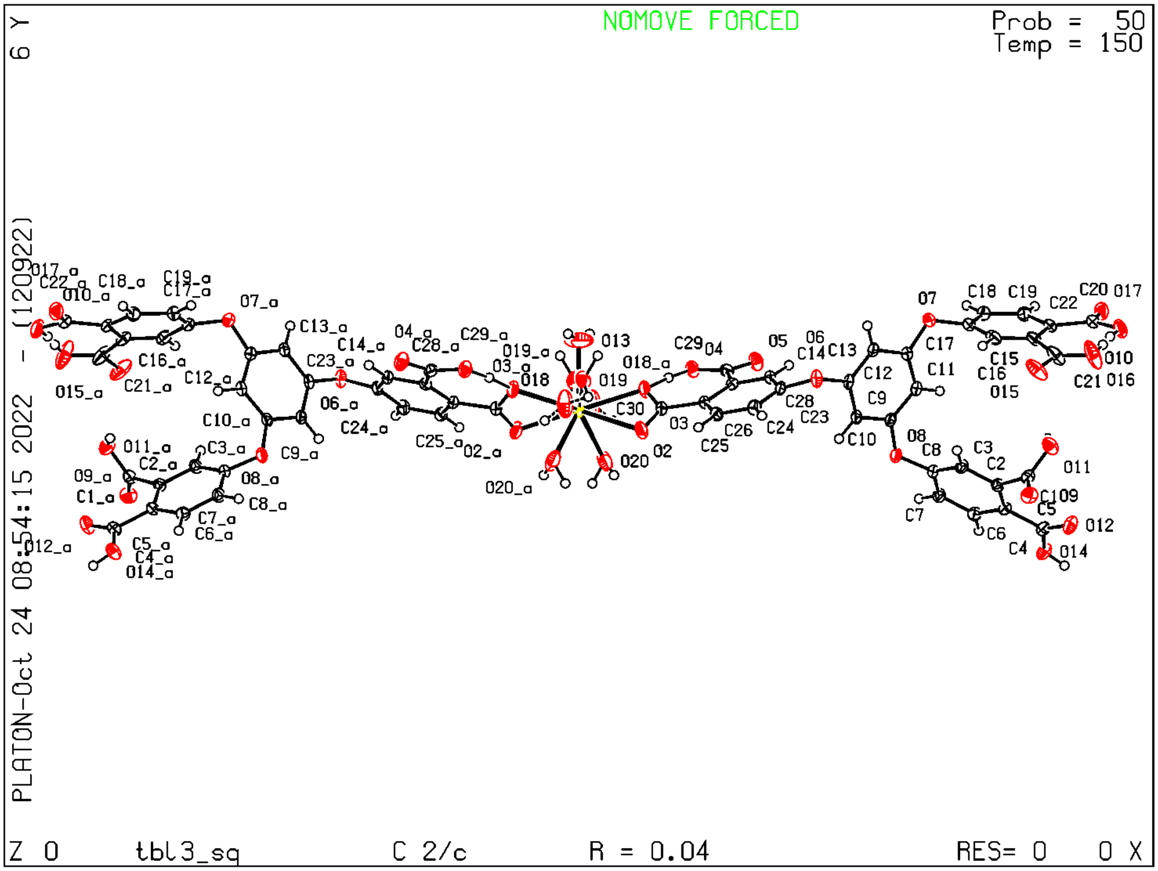

Supplement: Supplementary file 1 [file molecules-27-07849-s001.zip › Comp1_checkcif.pdf]
