# Supplementary material for: Variable Dimensionality of Europium(III) and Terbium(III) Coordination Compounds with a Flexible Hexacarboxylate Ligand
Source: Molecules. 2022 Nov 14;27(22):7849. doi: 10.3390/molecules27227849 (PMC9696389; doi:10.3390/molecules27227849)

checkCIF/PLATON report

Structure factors have been supplied for datablock(s) 1

THIS REPORT IS FOR GUIDANCE ONLY. IF USED AS PART OF A REVIEW PROCEDURE FOR PUBLICATION, IT SHOULD NOT REPLACE THE EXPERTISE OF AN EXPERIENCED CRYSTALLOGRAPHIC REFEREE.

No syntax errors found.   CIF dictionary   Interpreting this report

Datablock: 1

```
Bond precision:   C-C = 0.0048 A          Wavelength=0.71073

Cell:            a=8.6618(1)        b=10.0305(2)      c=19.2763(3)
                 alpha=87.992(1)    beta=80.183(1)    gamma=87.193(1)
Temperature:     150 K

                  Calculated                   Reported
Volume            1647.66(5)                   1647.66(5)
Space group       P -1                         P -1
Hall group        -P 1                         -P 1
Moiety formula    C30 H21 Eu O18, 4(H2 O)      C30 H21 Eu O18, 4(H2 O)
Sum formula       C30 H29 Eu O22               C30 H29 Eu O22
Mr                893.50                       893.49
Dx,g cm-3         1.801                        1.801
Z                 2                            2
Mu (mm-1)         2.000                        2.000
F000              896.0                        896.0
F000'             896.25
h,k,lmax          10,11,22                     10,11,22
Nref              5814                         5809
Tmin,Tmax         0.931,0.961                  0.685,0.747
Tmin'             0.852

Correction method= # Reported T Limits: Tmin=0.685 Tmax=0.747
AbsCorr = MULTI-SCAN

Data completeness= 0.999           Theta(max)= 25.000

R(reflections)= 0.0263( 5369)                         wR2(reflections)=
                                                      0.0565( 5809)

S = 1.036                  Npar= 503
```

The following ALERTS were generated. Each ALERT has the format
test-name_ALERT_alert-type_alert-level.
Click on the hyperlinks for more details of the test.

Alert level B

```
PLAT417_ALERT_2_B Short Inter D-H..H-D       H4B      ..H18A     .       2.07 Ang.
                                                   x,1+y,z  =      1_565 Check
```

Author Response: These alerts are all related to the hydrogen atoms of lattice water molecules.It is very difficult to locate hydrogen atoms accurately using X-ray data because these atoms have low scattering power. We have used two methods to add the hydrogen atoms of the water molecules, including DIF-Fourier maps and HADD instructions. The current results using HADD instructions are the best.

Alert level C

```
PLAT220_ALERT_2_C NonSolvent   Resd 1   O   Ueq(max)/Ueq(min) Range        3.3 Ratio
PLAT417_ALERT_2_C Short Inter D-H..H-D       H9A      ..H21A     .       2.13 Ang.
                                                   1+x,y,z  =      1_655 Check
```

Author Response: These alerts are all related to the hydrogen atoms of lattice water molecules.It is very difficult to locate hydrogen atoms accurately using X-ray data because these atoms have low scattering power. We have used two methods to add the hydrogen atoms of the water molecules, including DIF-Fourier maps and HADD instructions. The current results using HADD instructions are the best.

```
PLAT417_ALERT_2_C Short Inter D-H..H-D       H15      ..H18A     .       2.12 Ang.
                                                     x,y,z  =      1_555 Check
```

Author Response: These alerts are all related to the hydrogen atoms of lattice water molecules.It is very difficult to locate hydrogen atoms accurately using X-ray data because these atoms have low scattering power. We have used two methods to add the hydrogen atoms of the water molecules, including DIF-Fourier maps and HADD instructions. The current results using HADD instructions are the best.

```
PLAT417_ALERT_2_C Short Inter D-H..H-D       H18B     ..H21B     .       2.12 Ang.
                                                  x,-1+y,z  =      1_545 Check
```

Author Response: These alerts are all related to the hydrogen atoms of lattice water molecules.It is very difficult to locate hydrogen atoms accurately using X-ray data because these atoms have low scattering power. We have used two methods to add the hydrogen atoms of the water molecules, including DIF-Fourier maps and HADD instructions. The current results using HADD instructions are the best.

```
PLAT975_ALERT_2_C Check Calcd Resid. Dens.  0.70Ang From O21     .       0.68 eA-3
PLAT975_ALERT_2_C Check Calcd Resid. Dens.  0.72Ang From O21     .       0.61 eA-3
PLAT975_ALERT_2_C Check Calcd Resid. Dens.  1.05Ang From O4      .       0.49 eA-3
PLAT976_ALERT_2_C Check Calcd Resid. Dens.  0.58Ang From O21     .      -0.56 eA-3
PLAT976_ALERT_2_C Check Calcd Resid. Dens.  0.47Ang From O21     .      -0.55 eA-3
```

Alert level G

```
PLAT002_ALERT_2_G Number of Distance or Angle Restraints on AtSite          5 Note
PLAT003_ALERT_2_G Number of Uiso or Uij Restrained non-H Atoms ...          1 Report
PLAT004_ALERT_5_G Polymeric Structure Found with Maximum Dimension          1 Info
PLAT007_ALERT_5_G Number of Unrefined Donor-H Atoms .............          15 Report
PLAT154_ALERT_1_G The s.u.'s on the Cell Angles are Equal ..(Note)      0.001 Degree
PLAT176_ALERT_4_G The CIF-Embedded .res File Contains SADI Records          2 Report
PLAT186_ALERT_4_G The CIF-Embedded .res File Contains ISOR Records          1 Report
PLAT794_ALERT_5_G Tentative Bond Valency for Eu1       (III)     .       3.20 Info
PLAT860_ALERT_3_G Number of Least-Squares Restraints ............           8 Note
PLAT909_ALERT_3_G Percentage of I>2sig(I) Data at Theta(Max) Still        89% Note
PLAT910_ALERT_3_G Missing # of FCF Reflection(s) Below Theta(Min).          4 Note
PLAT933_ALERT_2_G Number of HKL-OMIT Records in Embedded .res File          3 Note
PLAT941_ALERT_3_G Average HKL Measurement Multiplicity ..........         3.6 Low
PLAT960_ALERT_3_G Number of Intensities with I  <   - 2*sig(I) ...          3 Check
PLAT967_ALERT_5_G Note: Two-Theta Cutoff Value in Embedded .res ..       50.0 Degree
PLAT978_ALERT_2_G Number C-C Bonds with Positive Residual Density.          6 Info
```

```
   0 ALERT level A = Most likely a serious problem - resolve or explain
   1 ALERT level B = A potentially serious problem, consider carefully
   9 ALERT level C = Check. Ensure it is not caused by an omission or oversight
  16 ALERT level G = General information/check it is not something unexpected

   1 ALERT type 1 CIF construction/syntax error, inconsistent or missing data
  14 ALERT type 2 Indicator that the structure model may be wrong or deficient
   5 ALERT type 3 Indicator that the structure quality may be low
   2 ALERT type 4 Improvement, methodology, query or suggestion
   4 ALERT type 5 Informative message, check
```

It is advisable to attempt to resolve as many as possible of the alerts in all categories. Often the minor alerts point to easily fixed oversights, errors and omissions in your CIF or refinement strategy, so attention to these fine details can be worthwhile. In order to resolve some of the more serious problems it may be necessary to carry out additional measurements or structure refinements. However, the purpose of your study may justify the reported deviations and the more serious of these should normally be commented upon in the discussion or experimental section of a paper or in the "special_details" fields of the CIF. checkCIF was carefully designed to identify outliers and unusual parameters, but every test has its limitations and alerts that are not important in a particular case may appear. Conversely, the absence of alerts does not guarantee there are no aspects of the results needing attention. It is up to the individual to critically assess their own results and, if necessary, seek expert advice.

Publication of your CIF in IUCr journals

A basic structural check has been run on your CIF. These basic checks will be run on all CIFs submitted for publication in IUCr journals (*Acta Crystallographica*, *Journal of Applied Crystallography*, *Journal of Synchrotron Radiation*); however, if you intend to submit to *Acta Crystallographica Section C* or *E* or *IUCrData*, you should make sure that full publication checks are run on the final version of your CIF prior to submission.

Publication of your CIF in other journals

Please refer to the *Notes for Authors* of the relevant journal for any special instructions relating to CIF submission.

PLATON version of 12/09/2022; check.def file version of 09/08/2022

Datablock 1 - ellipsoid plot

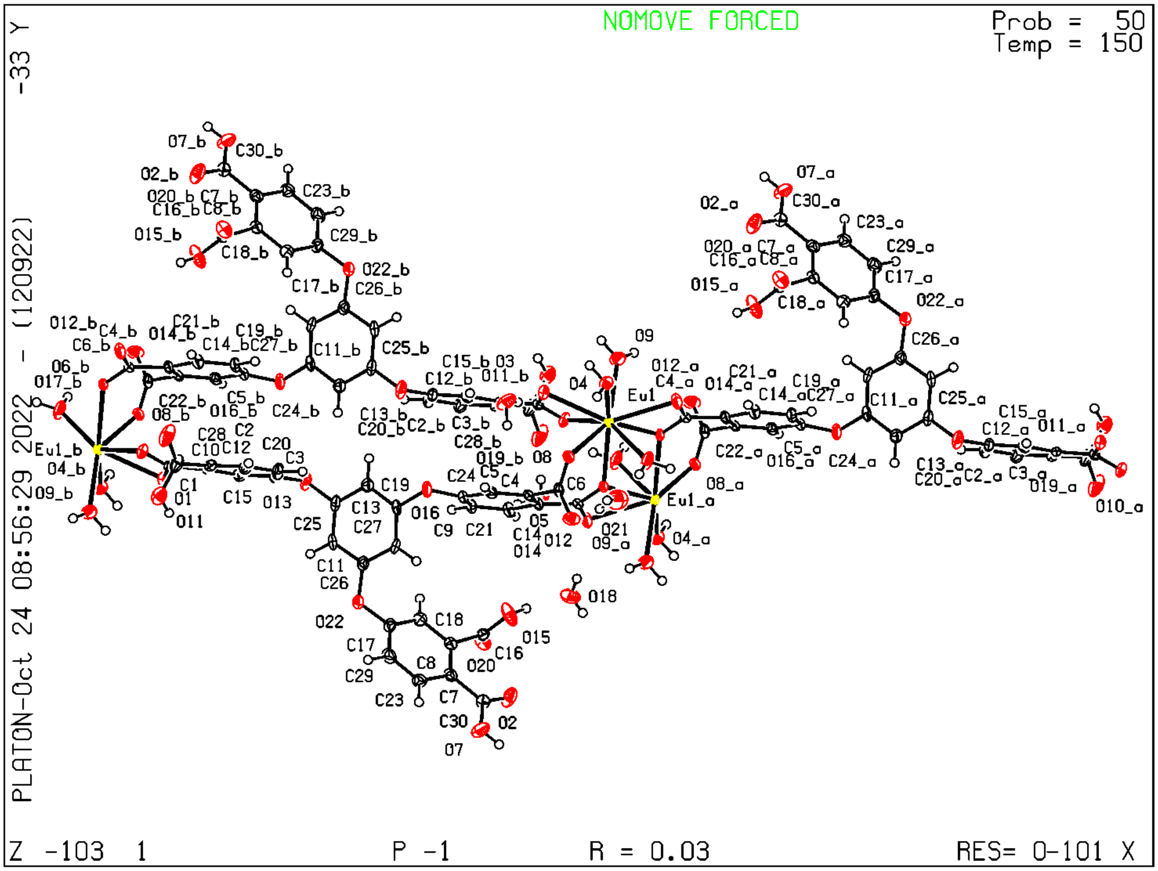

Supplement: Supplementary file 1 [file molecules-27-07849-s001.zip › Comp2_checkcif.pdf]
