# Supplementary material for: Variable Dimensionality of Europium(III) and Terbium(III) Coordination Compounds with a Flexible Hexacarboxylate Ligand
Source: Molecules. 2022 Nov 14;27(22):7849. doi: 10.3390/molecules27227849 (PMC9696389; doi:10.3390/molecules27227849)
Supplement: Supplementary file 1 [file molecules-27-07849-s001.zip › Supplementary materials.pdf]

SUPPLEMENTARY MATERIALS

Variable dimensionality of europium(III) and terbium(III) coordination compounds with a flexible hexacarboxylate ligand

Xiaolin Yu [1,2], Dmitry I. Pavlov [1], Alexey A. Ryadun [1], Andrei S. Potapov [1,*] and Vladimir P. Fedin [1,2,*]

[1] Nikolaev Institute of Inorganic Chemistry, Siberian Branch of the Russian Academy of Sciences, 3 Lavrentiev Ave., 630090, Novosibirsk, Russia

[2] Novosibirsk State University, 2 Pirogov Str. 630090, Novosibirsk, Russia

* Correspondence: potapov@niic.nsc.ru (A.S.P.); cluster@niic.nsc.ru (F.V.P.)

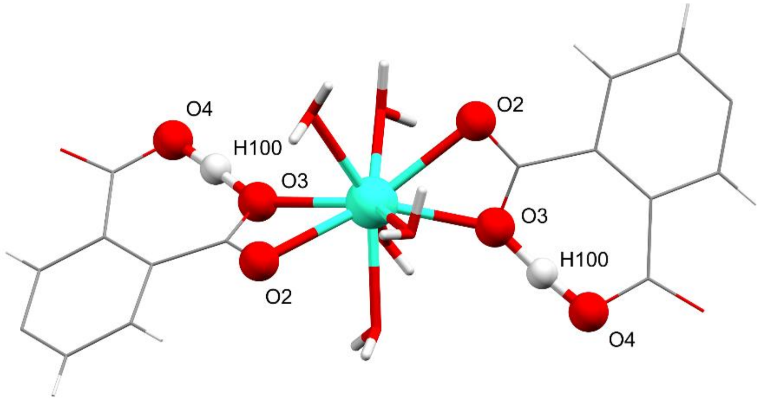

Figure S1. Fragment of the molecular structure of the compound **1** showing a hydrogen bond between the coordinated and uncoordinated carboxylic groups. The hydrogen atom shown as a gray sphere (H100) is disordered over two equivalent positions.

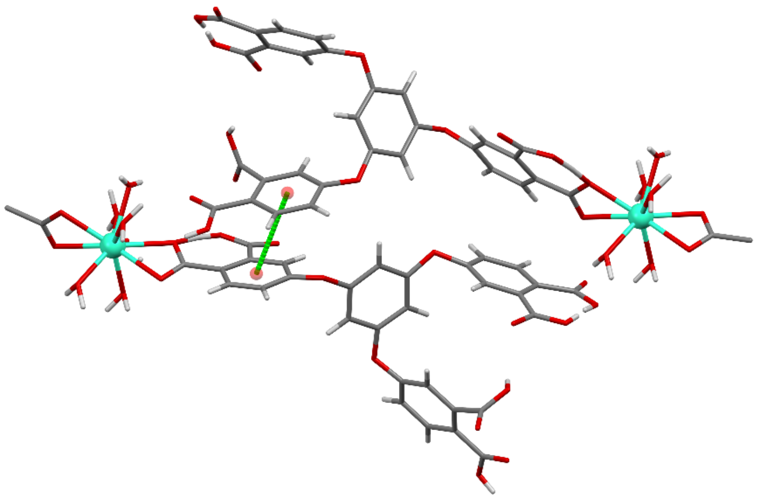

Figure S2. Fragment of the crystal structure of the compound **1** showing *π*–*π* interaction between the benzene rings of two molecules (green dashed line). For clarity, only one $H_{4.5}L^{1.5-}$ anion is shown in the coordination sphere of Tb^{3+}.

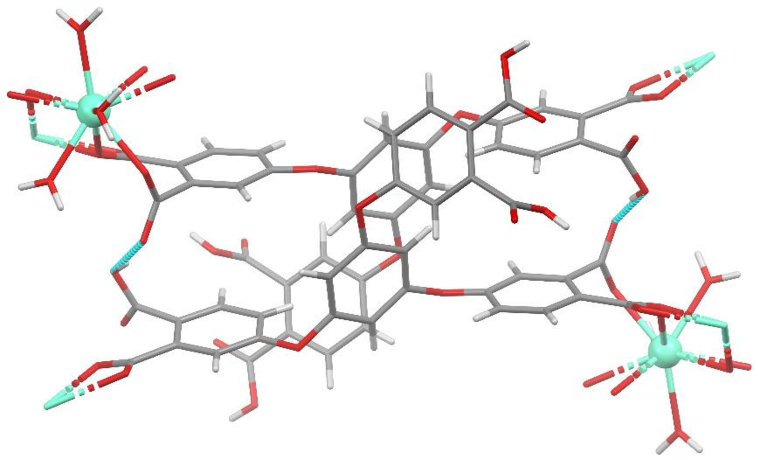

Figure S3. Fragment of the crystal structure of the compound **2** showing hydrogen bonds between the coordinated carboxylate and uncoordinated carboxylic groups in two different coordination polymer chains. View along the crystallographic axis *b*.

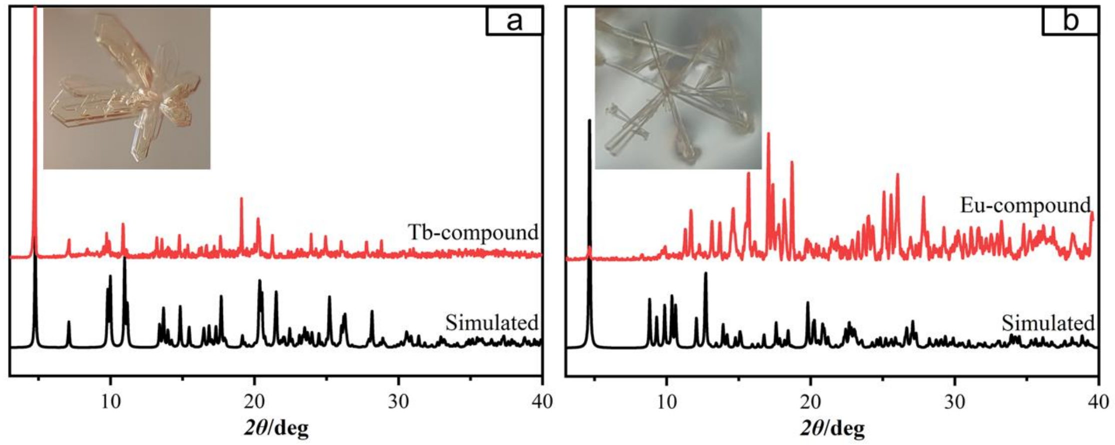

Figure S4. Calculated and experimental PXRD patterns of the compounds **1** (a) and **2** (b). Insets show photographic images of the single crystals of the corresponding compounds.

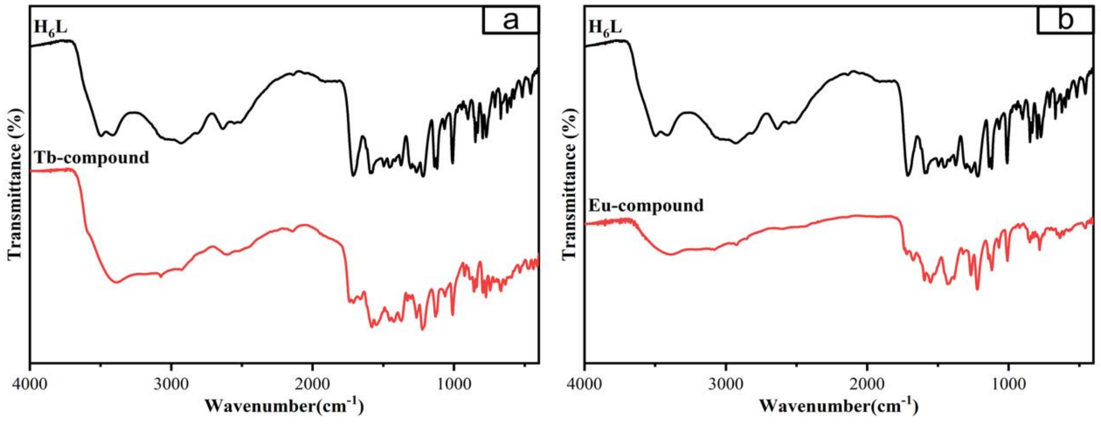

Figure S5. FT-IR spectra of H_6L ligand, compound **1** (a) and compound **2** (b).

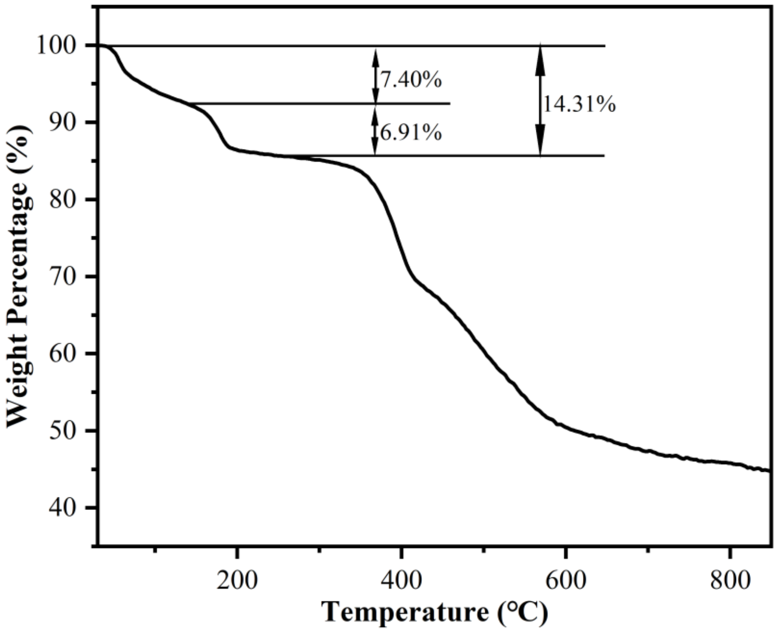

Figure S6. Thermogravimetric analysis curve for the compound **1**.

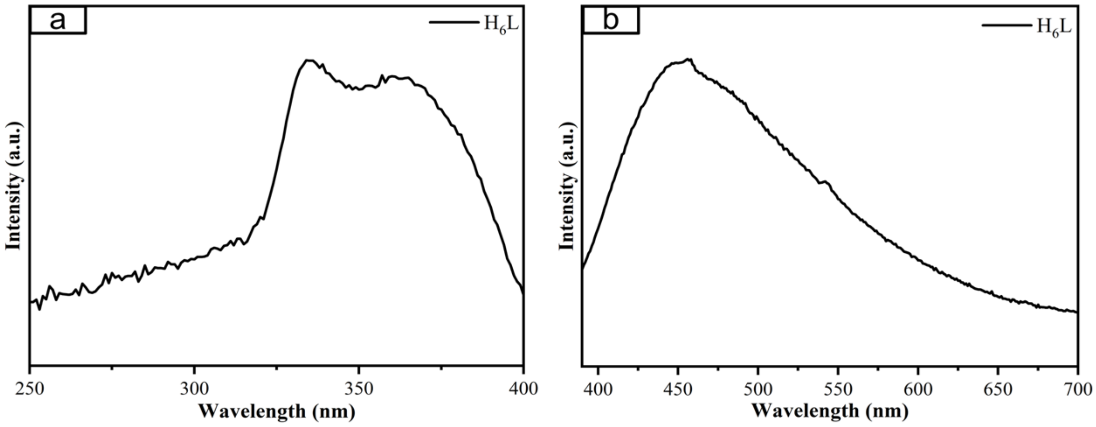

Figure S7. The solid-state excitation (λ_{em} = 450 nm) and emission (λ_{ex} = 370 nm) spectra of H_6L at room temperature.

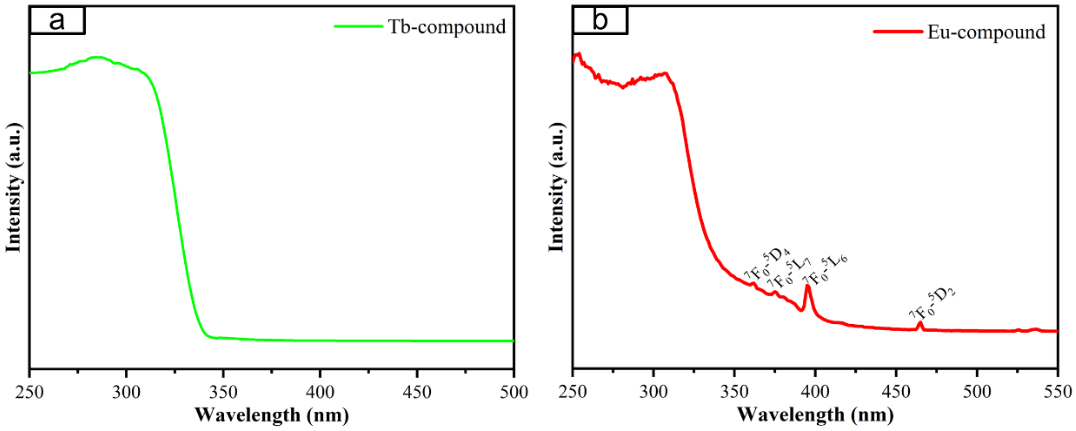

Figure S8. The solid-state excitation spectra of the compounds **1** (a, λ_{em} = 545 nm) and **2** (b, λ_{em} = 615 nm) at room temperature.

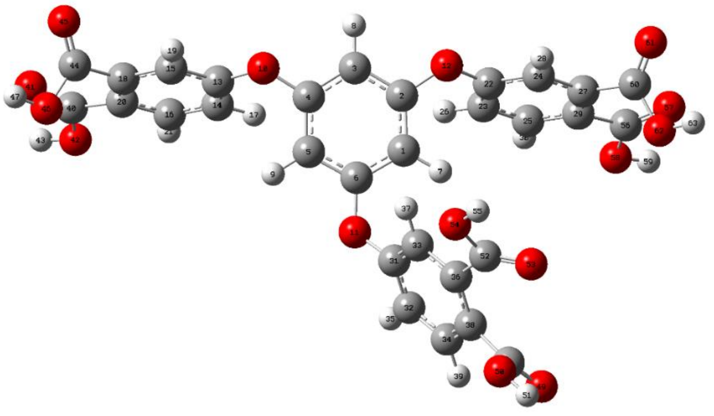

(**a**)

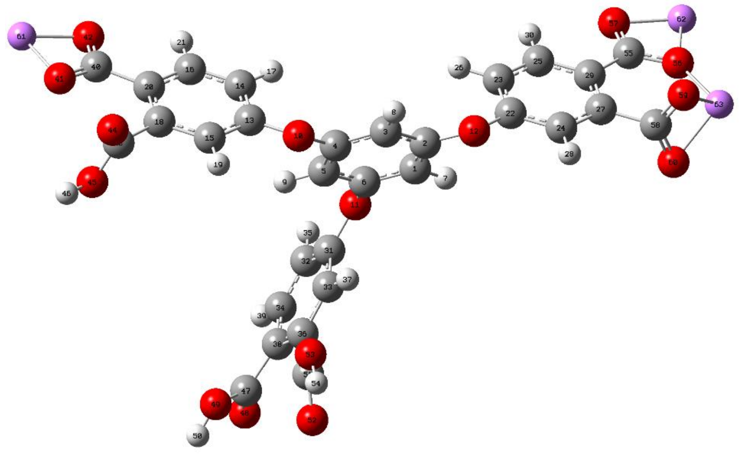

(**b**)

Figure S9. Optimized geometries of H_6L (**a**) and Li_3H_3L (**b**) obtained at B3LYP[GD3BJ] 6-31+G(d) level of theory.

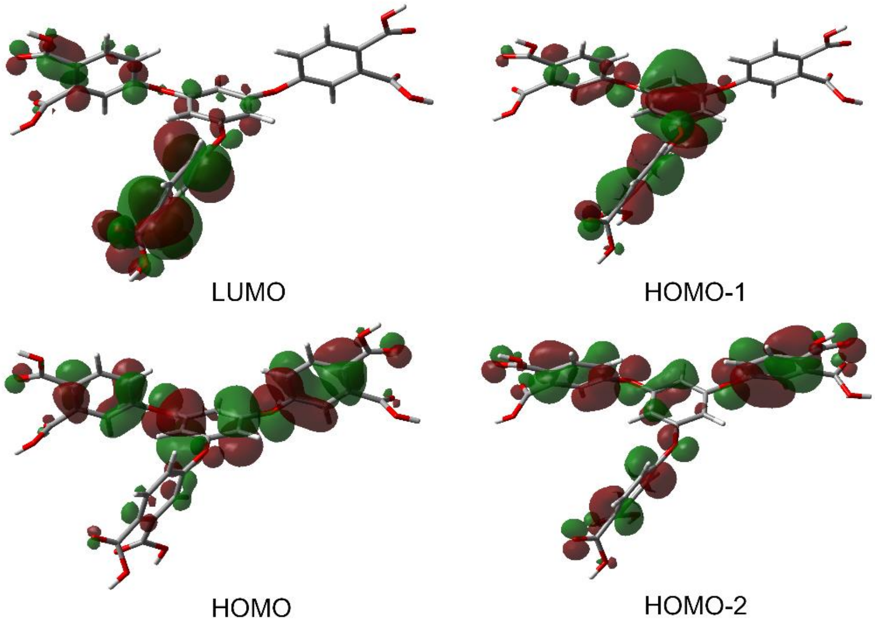

Figure S10. Isosurfaces (at 0.02 e/Bohr^3) of the molecular orbitals of H_6L ground state calculated at B3LYP[GD3BJ] 6-31+G(d) level of theory.

Table S1. Continuous shape measures criteria (S) for nine-coordinated metal centers in the compounds **1** and **2**.

Shape	Compound 1	Compound 2
Octagonal pyramid	21.087	21.677
Heptagonal bipyramid	18.050	16.760
Johnson triangular cupola J3	16.028,	13.170
Capped cube J8	10.952	9.331
Spherical-relaxed capped cube	9.966	8.153
Capped square antiprism J10	2.854	2.477
Spherical capped square antiprism	1.929	1.499
Tricapped trigonal prism J51	3.311	3.171
Spherical tricapped trigonal prism	2.226	2.239
Tridiminished icosahedron J63	13.304	12.804
Hula-hoop	10.779	10.386
Muffin	1.522	1.059

Table S2. Geometrical parameters of the hydrogen bonds in the compound **1**.

Hydrogen bond	d(D···A), Å	∠(D–H···A), °
O20–H···O16	3.290(3)	138(3)
O20–H···O10	2.720(3)	166(3)
O14–H···O15	2.627(3)	169(3)
O19–H···O12	2.844(3)	144(3)
O19–H···O15	2.759(3)	147(3)
O11–H···O4	3.096(2)	132(3)
O11–H···O5	2.593(2)	166(3)
C3–H···O14	3.441(3)	152(3)

Table S3. Geometrical parameters of the hydrogen bonds in the compound **2**.

Hydrogen bond	d(D···A), Å	∠(D–H···A), °
O9–H···O11	2.847(4)	171
O11–H···O14	2.556(4)	170
O2–H···O15	3.278(5)	172
O11–H···O20	3.467(4)	159
O29–H···O13	3.338(5)	141

Table S4. Calculated and experimental geometrical parameters of H_3L^{3-}.

Parameter[1]	Calculated[2]	Experimental[3]	Parameter[1]	Calculated[2]	Experimental[3]
d(C2–O12)	1.378	1.388	d(C29-C55)	1.493	1.509
d(C4–O10)	1.380	1.408	d(C55-O56)	1.305	1.255
d(C6–O11)	1.388	1.380	d(C55-O57)	1.271	1.256
d(C18–C43)	1.506	1.505	d(C36-C51)	1.496	1.509
d(C43-O44)	1.212	1.201	d(C51-O52)	1.213	1.195
d(C43-O45)	1.354	1.319	d(C51-O53)	1.357	1.311
d(C20-C40)	1.494	1.498	d(C38-C47)	1.494	1.489
d(C40-O41)	1.277	1.265	d(C47-O48)	1.215	1.208
d(C40-O42)	1.278	1.262	d(C47-O49)	1.353	1.307
d(C27-C58)	1.507	1.484	Θ(C4-O10-C13-C14)	39.0	31.8
d(C58-O59)	1.266	1.292	Θ(C6-O11-C31-C33)	157.9	141.8
d(C58-O60)	1.266	1.248	Θ(C2-O12-C22-C23)	37.6	134.4

[1] atom labels correspond to the calculated structure;
[2] from the geometry optimization of Li_3H_3L at B3LYP[GD3BJ] 6-31+G(d) level of theory;
[3] obtained from the X-Ray crystal structure of the compound **2**.

Table S5. Selected bond distances (d) and angles (φ) for the compound **1**.

**Bond**	**d, Å**	**Bond**	**d, Å**	**Bond**	**d, Å**
Tb01-O2	2.5473(16)	Tb01-O19	2.418(2)	Tb01-O3^{i}	2.3973(16)
Tb01-O3	2.3973(16)	Tb01-O20	2.330(2)	Tb01-O19^{i}	2.418(2)
Tb01-O13	2.380(3)	Tb01-O2^{i}	2.5473(16)	Tb01-O20^{i}	2.330(2)

**Angle**	**φ, °**	**Angle**	**φ, °**
O20-Tb01-O2^{i}	81.71(7)	O2_a-Tb01-O20^{i}	75.22(7)
O20-Tb01-O3^{i}	132.93(7)	O3_a-Tb01-O19^{i}	89.29(8)
O20-Tb01->O19^{i}	80.87(8)	O3_a-Tb01-O20^{i}	76.02(7)
O20-Tb01-O20^{i}	84.17(8)	O19_a-Tb01-O20^{i}	142.19(9)
O2_a-Tb01-O3^{i}	52.21(5)	Tb01-O2-C30	91.53(14)
O2_a-Tb01-O19^{i}	68.39(7)	Tb01-O3-C30	97.30(13)

Symmetry code: (i) −*x*+1, *y*, −*z*+3/2.

Table S6. Selected bond distances (d) and angles (φ) for the compound **2**.

**Bond**	**d, Å**	**Bond**	**d, Å**	**Bond**	**d, Å**
Eu1-O4	2.412(2)	Eu1-O9	2.425(3)	Eu1-O12^{i}	2.507(2)
Eu1-O6	2.506(2)	Eu1-O17	2.440(3)	Eu1-O1^{ii}	2.526(2)
Eu1-O8	2.385(2)	Eu1-O6^{i}	2.507(2)	Eu1-O10^{ii}	2.427(2)

**Angle**	**φ, °**	**Angle**	**φ, °**
O17-Eu1-O12^{i}	67.26(8)	O1^{ii} -Eu1-O10^{ii}	52.65(7)
O17-Eu1-O1^{ii}	138.28(8)	C1-O1-Eu1^{ii}	90.62(19)
O17-Eu1-O10^{ii}	147.18(9)	Eu1-O6-C6	134.25(19)
O6^{i}-Eu1-O12^{i}	51.95(7)	Eu1-O6-Eu1^{i}	112.53(8)
O6^{i}-Eu1-O1^{ii}	136.34(7)	C6-O6-Eu1^{i}	93.62(17)
O6^{i}-Eu1-O10^{ii}	87.52(7)	Eu1-O8-C22	120.99(19)
O12^{i}-Eu1-O1^{ii}	138.79(8)	C1-O10-Eu1^{ii}	95.13(18)
O12^{i}-Eu1-O10^{ii}	129.07(8)	C6-O12-Eu1^{i}	94.76(19)

Symmetry codes: (i) −*x*+2, −*y*+1, −*z*+1; (ii) −*x*+1, −*y*+1, −*z*+2.
